# Mitochondrial ROS Accumulation Contributes to Maternal Hypertension and Impaired Remodeling of Spiral Artery but Not IUGR in a Rat PE Model Caused by Maternal Glucocorticoid Exposure

**DOI:** 10.3390/antiox12050987

**Published:** 2023-04-24

**Authors:** Jing Long, Yan Huang, Gang Wang, Zhengshan Tang, Yali Shan, Shiping Shen, Xin Ni

**Affiliations:** 1 Department of Gynecology and Obstetrics, Xiangya Hospital Central South University, Changsha 410008, China; 2 National International Joint Research Center for Medical Metabolomics, Xiangya Hospital Central South University, Changsha 410008, China; 3 Reproductive Medicine Center, General Hospital of Southern Theatre Command, Guangzhou 510010, China; 4 Department of Physiology, Naval Medical University, Shanghai 200433, China

**Keywords:** glucocorticoids, preeclampsia, placenta, mitochondria, ROS, IUGR

## Abstract

Increased maternal glucocorticoid levels have been implicated as a risk factor for preeclampsia (PE) development. We found that pregnant rats exposed to dexamethasone (DEX) showed hallmarks of PE features, impaired spiral artery (SA) remodeling, and elevated circulatory levels of sFlt1, sEng IL-1β, and TNFα. Abnormal mitochondrial morphology and mitochondrial dysfunction in placentas occurred in DEX rats. Omics showed that a large spectrum of placental signaling pathways, including oxidative phosphorylation (OXPHOS), energy metabolism, inflammation, and insulin-like growth factor (IGF) system were affected in DEX rats. MitoTEMPO, a mitochondria-targeted antioxidant, alleviated maternal hypertension and renal damage, and improved SA remodeling, uteroplacental blood flow, and the placental vasculature network. It reversed several pathways, including OXPHOS and glutathione pathways. Moreover, DEX-induced impaired functions of human extravillous trophoblasts were associated with excess ROS caused by mitochondrial dysfunction. However, scavenging excess ROS did not improve intrauterine growth retardation (IUGR), and elevated circulatory sFlt1, sEng, IL-1β, and TNFα levels in DEX rats. Our data indicate that excess mitochondrial ROS contributes to trophoblast dysfunction, impaired SA remodeling, reduced uteroplacental blood flow, and maternal hypertension in the DEX-induced PE model, while increased sFlt1 and sEng levels and IUGR might be associated with inflammation and an impaired energy metabolism and IGF system.

## 1. Introduction

Preeclampsia (PE), which affects 2% to 8% of pregnancies worldwide, is one of the most common pregnancy-specific disorders clinically characterized by hypertension with proteinuria [1]. A theory is now generally accepted that PE progresses in two stages: (1) impaired spiral artery remodeling and abnormal placentation in the first trimester; followed by (2) the excessive release of antiangiogenic factors from the ischemic placenta that contributes to the maternal syndrome in later second and third trimesters [2,3]. However, PE has great heterogeneity due to various etiologies, leading to the molecular mechanisms underlying the pathogenesis being very different in PE with multiple etiologies. 

Glucocorticoids (GCs) are involved in many events during pregnancy, including embryo implantation and growth, placental development, and the initiation of parturition [4,5]. At term, maternal GCs levels rise to a 20-fold increase in mid-pregnancy concentrations, which is essential for pregnancy maintenance, fetal growth, and maternal adaptive responses [4]. Of note, the placenta and fetus can be exposed to increased concentrations of active GCs in the uterus through several ways, such as maternal stress, the impairment of maternal and placental GCs metabolism, and synthetic GCs administration due to medical requirements [5,6,7,8,9]. Maternal GCs levels have been higher in PE patients than in healthy pregnant women [10,11]. Since pregnant women may encounter various stressful stimuli, maternal GCs levels could occasionally increase during pregnancy. Thus, increased GCs levels could be a risk factor for PE pathogenesis. Our group and Zhang’s group have shown that maternal GCs exposure induces PE features in pregnant rats [11,12,13], which confirms that increased maternal GCs levels are involved in PE development and progress. Hence, elucidating the molecular mechanism underlying the pathogenesis and seeking a therapeutic strategy for PE with the etiology of maternal GCs exposure is of great interest. 

GCs exert their biological effects via binding with the GC receptor (GR). Classically, activated GR modulate the transcriptional activity of a large spectrum of targeted genes in the tissues [14,15]. In human placentas, GR are identified in various cell types, including extravillous trophoblasts (EVTs), cytotrophoblasts, and syncytiotrophoblasts [16,17,18,19,20]. GCs regulate the production and secretion of various bioactive factors, such as prostaglandins and corticotropin-releasing hormones in human placentas [21,22]. In rodent placentas, GR are identified in spongiotrophoblasts in the junction zone, syncytiotrophoblasts, and trophoblast giant cells in the labyrinth [23,24]. Although Zhang’s group [11] recently reported that reduced placental lipoxin A4 expression contributes to PE development in a rat model caused by maternal GCs exposure, several fundamental questions must be elucidated: (1) What molecular networks in placentas are affected by maternal GCs exposure? (2) Are there some common signaling pathways in the PE model caused by maternal GCs exposure and other PE models? (3) What are the potential intervention strategies and their effects on pregnancy outcomes? To address the above questions, we conducted experiments on the rat model of PE caused by dexamethasone (DEX) exposure during pregnancy. Unbiased transcriptomes coupled with metabolomics were applied to show the changed molecular network and enriched signaling pathways in response to DEX exposure during pregnancy. Morphological and functional experiments combined with cellular and molecular biology approaches were then conducted to elucidate the critical signaling pathways linked to the PE caused by maternal GCs exposure. MitoTEMPO, a mitochondria-targeted antioxidant, has been widely used to scavenge excess mitochondrial superoxide [25,26]. Since excess mitochondrial reactive oxygen species (mtROS) were found in the placentas, the effects of mitoTEMPO on PE development were studied, and underlying mechanisms were investigated by examining the association of PE features with molecular networks and key signaling pathways in the placentas. Finally, we investigated the roles of mitochondrial ROS in DEX regulation of human EVTs function in the cultured cell model. Our data gains new insights into the molecular mechanisms underlying the pathogenesis of PE with the etiology of maternal GCs exposure.

## 2. Materials and Methods

### 2.1. Animals

Sprague-Dawley rats aged 8–10 weeks (250–300 g) were purchased from SLAC Laboratory Animal Company, Hunan (Changsha, China). Animals were housed in 55% humidity, 25 °C, and 12-h day/night light cycle. All experimental procedures were supported by the Ethical Committee of Medical Research of Xiangya Hospital Central South University (Changsha, China). Female and male rats mated overnight and underwent vaginal smears the next morning. Gestation day (GD) 0.5 was calculated if large amounts of sperm were found on the smears. Dexamethasone-21-phosphate disodium salt (Sigma–Aldrich, St. Louis, MO, USA) was dissolved in saline to achieve the concentration of 0.39 mg/mL. In the first experiment, there were four groups. The pregnant rats were randomly divided into three groups (n = 6–8 in each group). They were subcutaneously administered either 0.13 mg/kg dexamethasone-21-phosphate disodium salt (equal to 0.1 mg/kg DEX), 0.26 mg/kg dexamethasone-21-phosphate disodium salt (equal to 0.2 mg/kg DEX), or vehicle (0.9% saline) once a day from GD7.5 to GD17.5. The nonpregnant female rats were divided into two groups. They were subcutaneously administered either 0.13 mg/kg dexamethasone-21-phosphate disodium salt or 0.9% saline once a day for 11 days. In the second experiment, the rats were administered dexamethasone-21-phosphate disodium salt (0.13 mg/kg) combined with mitoTEMPO (1 mg/kg, i.p.) once a day GD7.5 to GD17.5 (n = 8). MitoTEMPO (Sigma–Aldrich, St. Louis, MO, USA) was dissolved in saline. The dosage of DEX and mitoTEMPO was chosen based on our previous studies [13,27]. Blood pressure was measured in rats, as described previously [13,27]. Placental blood flow was detected by Doppler ultrasonography on GD19.5. On GD20.5, maternal blood was collected and rats were sacrificed after blood pressure measurement under deep anesthesia, and fetal, placental, and kidney tissues were collected. All qualified serum samples were used for subsequent ELISA assays.

### 2.2. Measurement of Blood Pressure

As previously mentioned, the mean arterial pressure (MAP) of each dam was measured every two days GD9.5-17.5 using the tail-cuff system (BP-300A automatic noninvasive blood pressure measurement system, Techmen software Ltd., Chengdu, China) [13,27]. Systolic blood pressure (SBP) was measured by right carotid artery intubation in pregnant rats at GD20.5, and recorded by the computer collecting and disposing of the system of organic signals (BL-420N, Techmen software Ltd., Chengdu, China). We successfully measured blood pressure in 5 rats treated with DEX, 5 rats treated with mitoTEMPO combined with DEX, and 8 control rats, and statistically analyzed all successful measurements.

### 2.3. Uteroplacental Blood Perfusion Measurement

As previously mentioned, utero-placental blood flow was measured by Doppler ultrasonography (Vevo 2100, FuJiFilm, Tokyo, Japan) on GD19.5 [27]. First, the rats were placed in an airtight box and anesthetized with 5% isoflurane. When they were anesthetized, the rats were removed and placed on a thermostatic operating blanket with a mask and sustained with 3% isoflurane through an anesthesia pump. After that, abdominal hairs were removed and gel was applied. Using a high frame rate 22 mHz probe attached to the abdomen, the instrument was switched to pulse mode to detect the waveform. Each pregnant rat was randomly measured at 2–4 implantation sites. The waveforms of spiral arteries (SA), maternal canal, and umbilical arteries (UmbA) were recorded, and the peak systolic velocity (PSV) was measured. Finally, the mean PSV of the SA, canal, and UmbA in each pregnant rat was obtained.

### 2.4. Measurement of Urinary Protein/Creatinine Concentration 

Urine protein/creatinine was measured as described in previous studies [27,28]. In brief, each pregnant rat was placed in a metabolic cage and fasted on GD18.5–19.5, and urine samples were obtained separately. Subsequently, concentrations of total urinary protein were measured by the Pierce^TM^ BCA Protein Test Kit (Thermo Scientific, Waltham, MA, USA). Creatinine concentrations were measured using the creatinine assay kit (Mlbio, Shanghai, China).

### 2.5. Pathological Assessment of the Kidney 

Kidney tissues were cut longitudinally, as previously described, and paraffin sections (3 µm) of the kidney were stained with hematoxylin-eosin (H and E) and periodocheveric acid (PAS) [13,27]. For each sample, 20 glomeruli were randomly selected for injury score, and its average value was taken. The main indicators observed were basement membrane thickening, capillary loop occlusion, and Bowman’s space narrowing. There are five grades: (1) Grade 0: normal; (2) Grade 1: capillary loop mild occlusion; (3) Grade 2: moderate capillary loop occlusion with mild basement membrane thickening; (4) Grade 3: capillary loop severe occlusion with basilar membrane thickening and Bowman’s space narrowing; (5) Grade 4: extremely severe capillary loop occlusion with thickening basement membrane and narrowing of Bowman’s space.

### 2.6. Pathological Immunostaining of Placental Tissue

As previously mentioned, paraffin sections of the placenta (5 µm) were used for subsequent detection [27]. Trophoblast invasion and SA remodeling were evaluated by immunohistochemistry of cytokeratin (Ck) and α-smooth muscle actin (α-Sma). The sections were incubated with antibodies against Ck (1:500; DAKO) and α-Sma (1:400; Servicebio, Wuhan, China) overnight at 4 °C, and the negative controls were given homologous IgG (DAKO) at the same concentration as the primary antibody. The vascular network in the labyrinth was examined by immunofluorescence of laminin. The sections were incubated with antibodies against laminin (Boster, Wuhan, China) overnight at 4 °C. The sections were then incubated with Alexa Fluor^®^ 594-conjugated secondary antibody (Abcam, Cambridge, UK) and counterstained with DAPI (Servicebio, Wuhan, China).

### 2.7. Electron Microscopy 

Fresh placental tissues were immersed in 2.5% glutaraldehyde solution and fixed for 24 h. The tissue was then post fixed with osmium tetroxide, dehydrated, and embedded in epoxy resin. Samples were sliced (50 nm), re-stained with uranyl acetate and lead citrate, and observed under a Hitachi H-7700 Transmission Electron Microscope (Hitachi, Tokyo, Japan). A blinded observer examined the morphology of mitochondria in the labyrinth zone. 

### 2.8. Enzyme-Linked Immunosorbent Assay (ELISA)

The concentrations of soluble fms-like tyrosine kinase 1 (sFlt1), soluble endoglin (sEng), prostaglandin E2 (PGE2) interleukin-1b (Il1b), and tumor necrosis factor α(Tnf-α) were determined using ELISA kits from ZCIBIO Technology Co., Ltd. (Shanghai, China).

### 2.9. Transcriptome Sequencing Analysis 

RNA-sequencing was conducted by Novogene Co., Ltd. (Beijing, China) as previously described [27]. Briefly, the fragments per kilobase of exon model per million mapped fragments (FPKM) of each gene were calculated. Gene differential expression analysis, heatmap, volcano plot, and pathways enrichment analysis were performed on the NovoMagic (https://magic.novogene.com/ (accessed on 25 April 2022)). Differential genes were defined by an adjusted *p*-value < 0.05 and fold change >1.2. The STRING (https://string-db.org/ (accessed on 13 May 2022)) database was used to construct the protein-protein interaction (PPI) network. 

### 2.10. Targeted Metabolomics

Targeted central carbon metabolomics was conducted by Novogene Co., Ltd. as previously described [27]. Peak areas represent the relative content of the corresponding metabolites, and were used for statistical analysis.

### 2.11. Cell Culture and In Vitro Trophoblast Migration and Invasion Assessment

HTR-8/SVneo (HTR8), an EVTs cell line, was a gift from Prof. Charles H. Graham (Queen’s University, Canada). The cells were grown in HAM F12/DMEM (Hyclone) supplemented with 10% FCS, 100 UI/mL penicillin, and 100 mg/mL streptomycin at 37 °C in 5% CO_2_ and 95% air. The cells were treated with various concentrations of DEX in the absence and presence of mitoTEMPO for 24 h, and the cells were collected.

As described previously, HTR8 migration function was examined in the 8-μm pore Boyden chamber (Costar, Corning Inc., Corning, NY, USA) [13,27]. Cells were seeded into the upper chamber and allowed to migrate into the bottom chamber in the media containing various concentrations of DEX with or without mitoTEMPO. After 24 h incubation, the cells that moved to the underside of the membrane were stained with DAPI (Beyotime Biotechnology, Shanghai, China). The number of cells from 3 random fields was counted under the microscope (at ×200 magnification). 

Cell invasion was assessed by the ability of cells to digest and invade the Matrigel-coated 8 μm pore size polycarbonate membrane Transwell inserts (Costar), as previously described. Briefly, HTR8 cells were seeded in the inserts and treated with DEX in the presence and absence of mitoTEMPO for 24 h. Cells that did not invade the submembrane were scraped and then fixed, and stained by DAPI. Three fields were randomly selected under the microscope and counted (×200 magnification). 

### 2.12. Assay of Mitochondrial Oxygen Consumption

The mitochondrial oxygen consumption rate (OCR) and extracellular acidification rate (ECAR) were measured in a Seahorse XFe96 Flux Analyzer (Seahorse Biosciences, Agilent, Santa Clara, CA, USA). Briefly, the cells (5000/well) were plated in quadruplicate in XFe96 extracellular flux assay plates and incubated at 37 °C with 5% CO_2_. The day before the test, the XF calibration kit was incubated at 37 °C overnight to equilibrium. Next, the medium was changed to contain 5 mM sodium pyruvate (Thermo Scientific, Waltham, MA, USA), 10 mM glucose (Thermo Scientific, Waltham, MA, USA), and 2 mM glutamine and was incubated for 1 h before determination. OCR was monitored by adding 1 μM oligomycin, 1 uM FCCP, and 1 uM antimycin A (Seahorse XF Cell Mito Stress Test Kit, Agilent, Santa Clara, CA, USA). ECAR was monitored by adding 10 mM glucose, 1 mM oligomycin, and 50 mM 2-deoxyglucose (2-DG) (Seahorse XF Glycolysis Stress Test Kit, Agilent, Santa Clara, CA, USA). The test values normalized by protein content (ug) and are shown as the OCR (pmol O_2_/min/ug protein) and the ECAR (mPH/min/ug); the various parameters of OCR were calculated as basal respiration and ATP production, and the ECAR was calculated as glycolytic capacity and glycolytic reserve.

### 2.13. Mitochondrial Isolation and Function Determination

Mitochondria of placental tissues and cells were isolated according to the kit’s instruction (Beyotime). MtROS level was assessed using the MitoSOX^TM^ Red mitochondrial superoxide indicator (Invitrogen, Waltham, MA, USA). ATP level and mitochondrial membrane potential (MMP) were measured by an enhanced ATP Assay Kit (Beyotime), and an enhanced MMP assay kit with JC-1 (Beyotime) according to manufacturer instructions, respectively. 

### 2.14. Total RNA Extraction and Quantitative Real-Time PCR (Q-PCR)

RNAs were extracted by TRIzol reagent (Accurate Biotechnology, Hunan, China), and reverse transcription of 1µg RNA was performed using PrimeScript RT Master Mix Kit (TaKaRa Bio. Inc., Dalian, China) to generate cDNA. Primers (Table S1) were synthesized by Tsingke Biotechnology (Beijing, China). The reaction system containing 2.0 μL diluted cDNA, 0.2 μM primers per pair, and 1×ChamQ Universal SYBR qPCR Master Mix (Vazyme Biotechnology, Nanjing, China) was formulated, and was performed on the Real-Time PCR Detection System (BioRad, Hercules, CA, USA). After the detection, the specificity of primers was evaluated by the melting curve. β-actin was used as an internal control and the relative quantification of the target gene was assessed by arithmetic formulas (2^−ΔΔCt^).

### 2.15. Mitochondrial DNA Copy Number Detection

MtDNA copy number was determined as described above [29]. In short, genomic DNA was extracted from placental tissue or cells using the Universal Genomic DNA Kit (TaKaRa Bio. Inc., Dalian, China). Then, mtDNA copy number was evaluated by qPCR using mitochondria-specific 16SrRNA as the target gene, the ribosomal protein s18 (RPS18) was used in rats, and nuclear β-2 microglobulin (B2M) was used in human cells as internal controls. Primers are listed in Table S1.

### 2.16. Western Blotting Analysis 

For placental tissues, about 20–30 mg tissue was weighed into a centrifuge tube, and 300 μL RIPA (Beyotime, Shanghai, China) containing PMSF (Beyotime, Shanghai, China), protease inhibitor (Topscience Co., Ltd. Shanghai, China); phosphatase inhibitor (Topscience Co., Ltd. Shanghai, China) was added for cracking, steel balls were added, then the centrifuge tube was placed on a pre-cooled grinding rack and homogenized in the grinder at a frequency of 60 Hz, 30 s/5 times. For cells, 100 μL RIPA containing PMSF, protease inhibitor, and phosphatase inhibitor was added to each well of 6-well plates for protein cleavage. The cells were rapidly shaken at 4 °C for 15 min, and then the lysate was collected. Next, protein concentration was determined by the Pierce^TM^ BCA Protein Test Kit (Thermo Scientific, MA, USA), and protein samples were prepared by loading buffer. The same amount of protein (30 micrograms) was then separated by SDS-PAGE electrophoresis and transferred to the PVDF membranes (Merck Millipore, Burlington, MA, USA). After incubation with blocking buffer, the membranes were incubated with the corresponding primary antibody at 4 °C overnight. Primary antibodies are listed in Table S2. The secondary antibodies were then incubated, and finally the bands were developed through the Tanon 4600SF Image system (Tanon, Shanghai, China) and using the enhanced chemiluminescence western blotting detection system (Vazyme Biotechnology, Nanjing, China). Subsequently, Image J was used for the semi-quantification of bands density. The target bands were quantified as the relative protein level by the ratio of density to β-actin.

### 2.17. Statistical Analysis

GraphPad-Prism9 was used for graphic presentation and statistical analysis. Results are represented as the mean ± standard deviation (SD). Normal distribution was evaluated by the Shapiro-Wilk test. Statistical significance was determined by sample distribution and homogeneity of variance. Statistical comparisons between two groups were determined by the two-tailed Student’s *t* test. For multiple comparisons among three or four groups, one-way ANOVA was applied. *p* < 0.05 was considered statistically significant.

## 3. Results

### 3.1. DEX Induces PE-like Features and Results in Changes in a Large Spectrum of the Transcriptome in Placentas of Pregnant Rats

**Figure 1:**
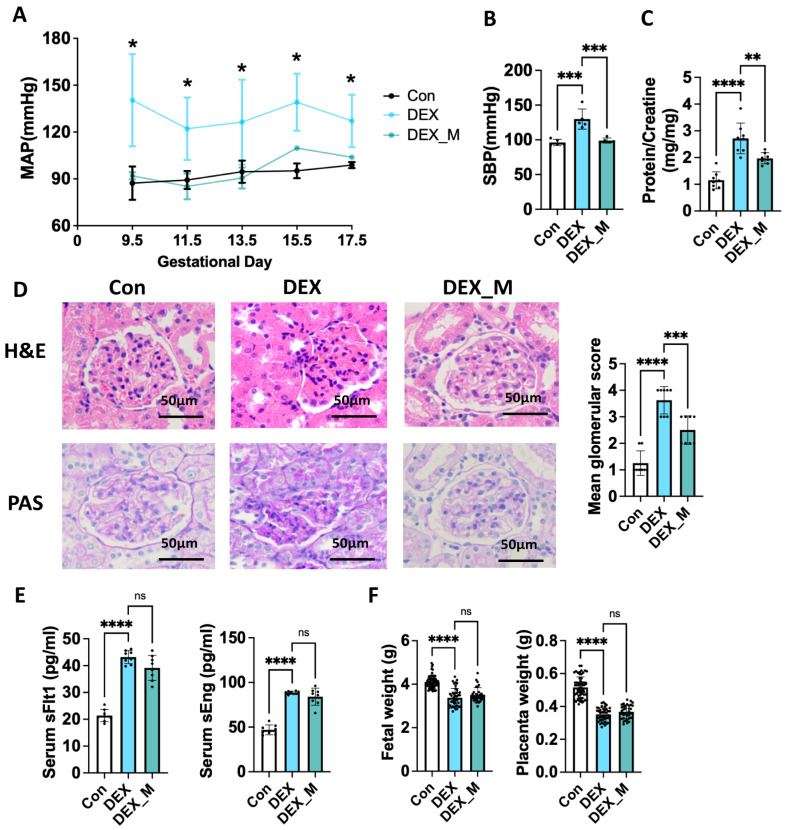
Maternal DEX exposure leads to PE-like features in pregnant rats and therapeutic effects of mitoTEMPO on PE-like features caused by DEX. Pregnant rats were administered DEX, DEX combined with mitoTEMPO, or saline from GD7.5 to GD17.5. Urine was collected from GD18.5 to GD19.5. The rats were sacrificed on GD20.5 for the collection of blood and tissues. (**A**) MAP was measured from GD 9.5 to GD 17.5. (**B**) SBP was measured on GD20.5. (**C**) Urine protein/creatinine (mg/mg). (**D**) H and E and PAS staining were used to observe glomerular morphology. Left panel: the representative images (400×). Right panel: Pathological score of glomeruli. (**E**) Circulatory sFlt1 and sEng levels in pregnant rats on GD20.5. (**F**) Individual fetal and placental weight. Fetal and placental weight from 8 dams of each group were measured on GD 20.5. * *p*  <  0.05, ** *p*  <  0.01, *** *p*  <  0.001, **** *p*  <  0.0001, ns: no significance. Con: control; DEX_M: DEX combined with mitoTEMPO treatment. H and E: Hematoxylin and Eosin; PAS: Periodic acid-Schif.

As expected, DEX treatment could lead to PE-like features in pregnant rats. As shown in Figure 1, mean arterial pressure (MAP) in pregnant rats with DEX treatment (0.1 mg/kg) was significantly elevated compared with control rats. Notably, MAP was elevated as early as GD 9.5 (Figure 1A). A significant increase in systolic blood pressure (SBP) measured on GD 20.5 was found in the DEX group (Figure 1B). In addition, the protein/creatinine in urine was increased (Figure 1C). Histology analysis of the kidney showed mesangial hypercellularity, capillary loops occlusion, and the glomeruli’s urinary space (Figure 1D). Circulatory levels of sFlt-1 and sEng were significantly elevated in DEX rats (Figure 1E). Intrauterine growth restriction (IUGR) was found in the DEX group, as evidenced by decreased fetal and placenta weights (Figure 1F). Treatment of the pregnant rats with DEX at 0.2 mg/kg could also increase blood pressure and IUGR (Supplemental Figure S1A,B). However, treating nonpregnant female rats with DEX at 0.1 mg/kg did not affect blood pressure, renal function, or morphology (Supplemental Figure S1C–E). 

**Figure 2:**
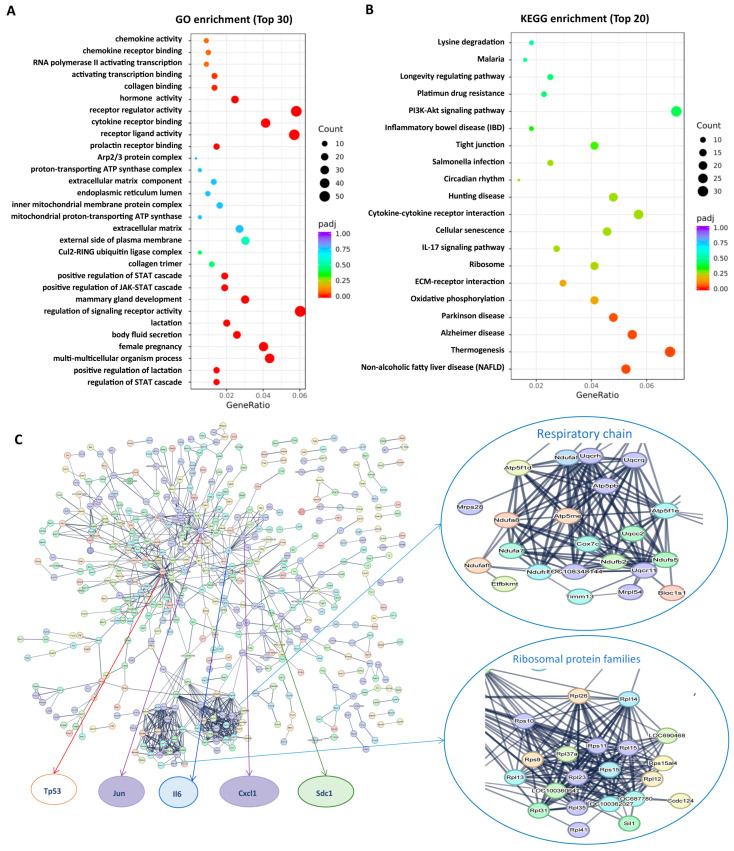
DEX resulted in changes in a large spectrum of the transcriptome in the placentas of pregnant rats. Pregnant rats were administrated with DEX or saline from GD7.5 to GD17.5. The rats were sacrificed on GD20.5. (**A**) GO enrichment analysis (TOP 30) of RNA-seq of placentas. (**B**) KEGG enrichment analysis (TOP 20) of RNA-seq of placentas. (**C**) PPI network for DEGs. PPI network was obtained using the STRING database with high confidence (interaction score > 0.7). Disconnected nodes have been ruled out. Con: control.

To better understand the placental molecular network affected by DEX exposure, RNA-seq was performed. The cluster heatmap showed a significant difference in transcriptome characteristics between the DEX and control groups (Supplemental Figure S2A). The DEX group presented 610 upregulated genes and 578 downregulated genes compared to the control group (Supplemental Figure S2B). Gene Ontology (GO) enrichment showed that many signaling pathways, including chemokine activity, cytokine receptor binding, positive regulation of JAK-STAT cascade, STAT cascade, and ATP synthase complex were significantly enriched (Figure 2A). In contrast, the Kyoto Encyclopedia of Genes and Genomes (KEGG) analysis showed that cytokine-cytokine receptor interaction, tight junction, and oxidative phosphorylation (OXPHOS) were significantly enriched (Figure 2B). 

Further, we constructed a Protein-Protein Interaction (PPI) network for differential genes (DEGs) using the STRING database (Figure 2C) with high confidence (interaction score > 0.7). After hiding disconnected nodes had been ruled out, the network comprised 998 nodes and 922 edges. There were several hub genes, including OXPHOS genes (zoomed in Figure 2C, ribosomal protein families, Tp53, Jun, interleukin 6 (Il6), C-X-C motif chemokine ligand 1 (Cxcl1), Syndecan 1 (Sdc1), etc. (zoomed in Figure 2C). 

### 3.2. Placental Mitochondrial Function and Morphology Are Impaired, and Mitochondrial ROS Production Is Significantly Increased in the DEX-Induced PE Model

**Figure 3:**
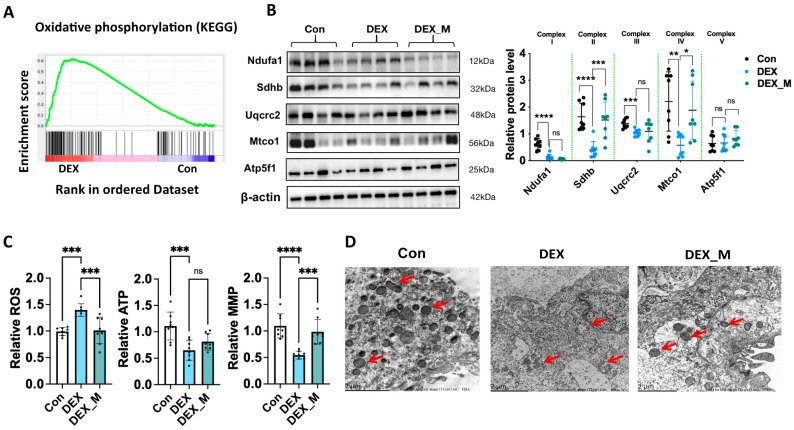
The effects of DEX and DEX combined with mitoTEMPO on Placental OXPHOS complexes, mitochondrial function, and morphology in pregnant rats. Pregnant rats were administrated with DEX, DEX combined with mitoTEMPO, or saline from GD7.5 to GD17.5. The rats were sacrificed on GD20.5. The placental tissues were collected for the determination of mitochondrial function and morphology, and expression levels of complexes in OXPHOS. (**A**) GSEA analysis showed the signaling related to OXPHOS in DEX rats. (**B**) Protein levels of the factors in each respiratory chain complex. Left panel: representative blotting images of OXPHOS complexes. Right panel: analysis of cumulative data of relative protein level. (**C**) Mitochondrial ROS, ATP and MMP. (**D**) Electron microscope analysis of placental tissues. Representative images of the electron microscope (10,000×). Red arrow: mitochondria. * *p*  <  0.05, ** *p*  <  0.01, *** *p*  <  0.001, **** *p*  <  0.0001, ns: no significance. Con: control.

Given that OXPHOS genes were hub genes in the transcriptome, we further analyzed the OXPHOS gene programs. Figure 3A showed OXPHOS enrichment in gene set enrichment analysis (GSEA). Figure 3B showed that the protein levels of NADH: ubiquinone oxidoreductase subunit A1 (Ndufa1) in complex I, succinate dehydrogenase complex iron sulfur subunit B (Sdhb) in complex II, ubiquinol-cytochrome C reductase core protein 2 (Uqcrc2) in complex III, and mitochondrially encoded cytochrome C oxidase I (Mtco1) in complex IV were decreased, while ATP synthase peripheral stalk-membrane subunit B (Atp5f1) in complex V was not significantly changed in the DEX group, suggesting that mitochondrial respiratory chain (i.e., electron transport chain) components I, II, III, and IV be at least affected in DEX-induced PE-like model. 

Next, we examined mitochondrial functions, including mtROS, ATP and mitochondrial membrane potential (MMP) level, and mitochondrial morphology. As shown in Figure 3C, ATP level and MMP were significantly decreased, while mtROS production was significantly increased in DEX-induced PE rats. Transmission electron microscope analysis showed a decreased number of mitochondria, morphological shrinkage, abnormal cristae, and significant alterations in the matrix structure and membrane in placentas of DEX-induced PE rats (Figure 3D). 

### 3.3. Scavenging mtROS Significantly Reverses Maternal Hypertension and Improves Renal Damage but Not IUGR and Elevated Circulatory sFlt-1 and sEng Levels in DEX-Induced PE Rats

Given that excess mitochondrial ROS production occurred, we used mitoTEMPO, a mitochondrial superoxide scavenger, to explore its curative effect on the DEX-induced PE model. As shown in Figure 3, mitoTEMPO treatment reversed elevated blood pressure (MAP and SBP) in DEX rats from GD9.5 to GD17.5 (Figure 1A,B). It also reduced protein/creatinine in urine and partly improved renal injury score caused by DEX (Figure 1C,D), suggesting that mtROS accumulation contributes to maternal hypertension. However, the renal injury score of DEX combined with mitoTEMPO treatment was higher than control rats. MitoTEMPO did not significantly improve the weight of the fetus and placenta, or the circulatory sFlt1 and sEng levels in DEX rats (Figure 1E,F). 

### 3.4. Scavenging mtROS Significantly Partly Improves the Expression of the Factors in OXPHOS, Mitochondrial Function and Morphology in DEX Rats 

As shown in Figure 3B, mitoTEMPO treatment could reverse decreased Sdhb and Mtco1 protein levels caused by DEX, but not the DEX-induced reduction of Ndufa1 and Uqcrc2 levels, which suggests that reduced Sdhb and Mtco1 protein levels in the DEX group might be attributed to excess ROS production. 

Functional studies showed that mitoTEMPO treatment reduced ROS production and increased MMP level, but did not affect ATP production, suggesting that mitoTEMPO treatment partly reverses mitochondrial function (Figure 3C). The morphological analysis also showed that mitochondrial morphology was improved, as evidenced by transparent cristae andmatrix structure (Figure 3D). 

The above data indicate that DEX primarily suppresses Ndufa1 and uqcrc2 expression, and subsequently leads to the accumulation of ROS, which contributes to mitochondrial dysfunction and abnormality of mitochondrial morphology. 

### 3.5. MtROS Accumulation Contributes to Impaired SA Remodeling, Uteroplacental Blood Flow and Placental Hypoxia but Not Fetal Blood Flow in DEX-Induced PE Rats

**Figure 4:**
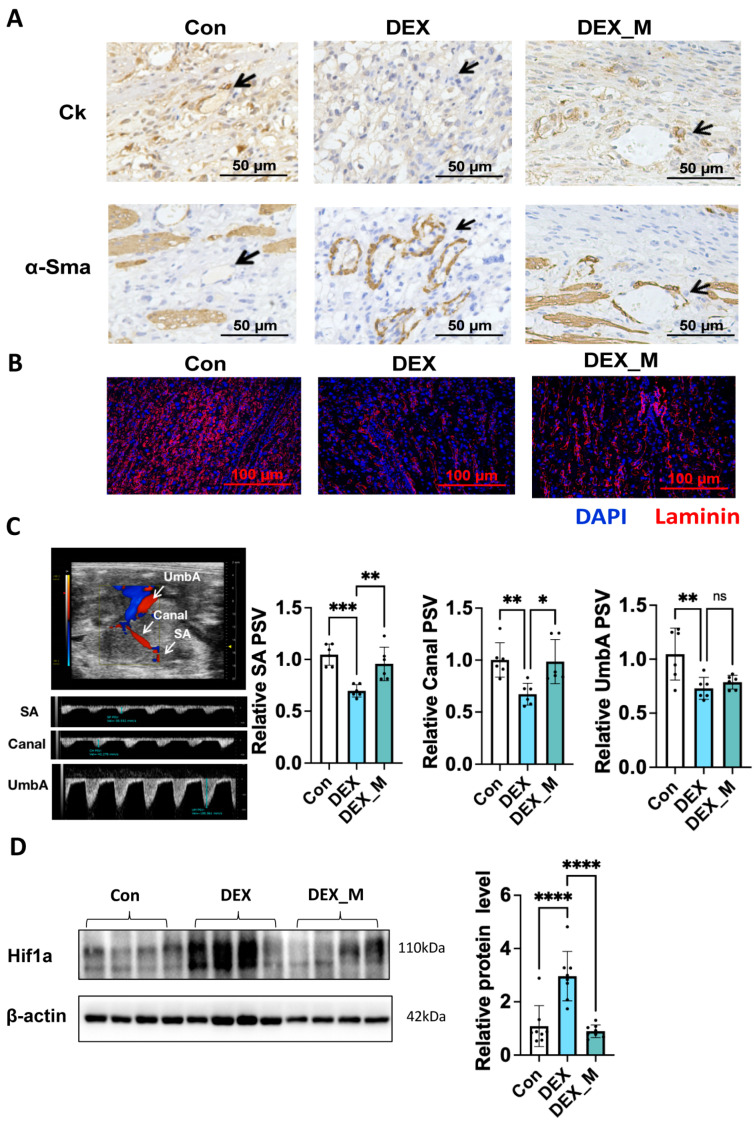
MitoTEMPO significantly alleviated impaired SA remodeling, uteroplacental blood flow and placental hypoxia, but not fetal blood flow in DEX-induced PE rats. (**A**) Placental SA remodeling. Representative images of Ck and α-Sma staining in SA (200×). Black arrow: SA. (**B**) Placental vasculature network. Representative immunofluorescence images of laminin (100×). (**C**) Doppler ultrasonography. Left panel: the representative full view and pulse waveform of placental vessels, SA and canal of the maternal side, UmbA of the fetal side. Right panel: cumulative PSV of SA, Canal, and UmbA. (**D**) Left panel: representative blotting image of Hif1a. Right panel: analysis of cumulative data of relative protein level. * *p*  <  0.05, ** *p*  <  0.01, *** *p*  <  0.001, **** *p*  <  0.0001, ns: no significance. Con: control; DEX_M: DEX combined with mitoTEMPO treatment.

As mentioned, normal placentation is associated with proper SA remodeling. As shown in Figure 4A, higher α-Sma staining intensity and less trophoblast infiltration in SA were displayed in the DEX group compared with the control group, indicating that DEX impaired SA remodeling. MitoTEMPO treatment could partly reverse SA remodeling, as evidenced by increased Ck staining and decreased α-Sma staining (Figure 4A). Laminin staining showed that the branching of vasculature in the labyrinth zone was reduced in the DEX group compared with controls, and mitoTEMPO could improve the vasculature network in the labyrinth zone (Figure 4B). However, the vascular branching in DEX combined with the mitoTEMPO treatment group was still less than in the control group.

DEX exposure led to reduced uteroplacental blood flow, as evidenced by a significant reduction in the peak systolic velocity (PSV) of SA and the maternal canal (Canal), and reduced fetal blood flow; i.e., reduced PSV of umbilical artery (UmbA) compared with the control group (Figure 4C). MitoTEMPO treatment recovered the PSV of SA and the Canal, but not the PSV of UmbA (Figure 4C). Figure 4D showed that placental ischemia occurred in DEX rats as evidenced by the increased hypoxia inducible factor-1A (Hif1a) level in DEX rats compared to control rats, which was reversed by mitoTEMPO treatment. These data suggest that placental ischemia caused by DEX is improved by mitoTEMPO treatment. 

### 3.6. MitoTEMPO Treatment Reverses Some Pathways Including OXPHOS and Glutathione Pathways and Improves mtDNA Copy in DEX-Induced PE Rats 

**Figure 5:**
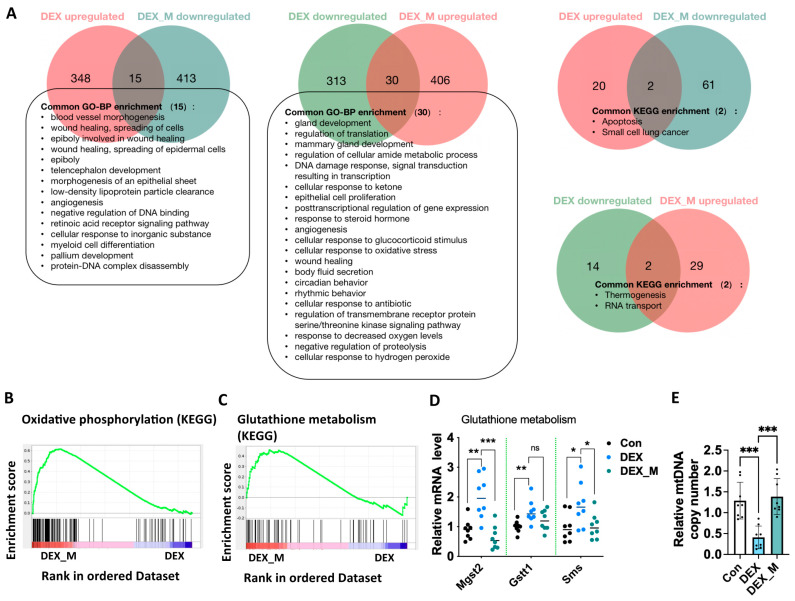
MitoTEMPO treatment reversed some pathways including OXPHOS and glutathione pathways, and improved mtDNA in DEX−induced PE model. (**A**) Venn diagram of GO−BP and KEGG pathways based on the transcriptomic data from Con, DEX, and DEX_M. groups. (**B**) GSEA showed the signaling related to OXPHOS. (**C**) GSEA analysis results of signaling related to glutathione metabolism. (**D**) Analysis of cumulative Q-PCR data of glutathione metabolism−related genes. (**E**) mtDNA copy number. * *p*  <  0.05, ** *p*  <  0.01, *** *p*  <  0.001, ns: no significance. Con: control; DEX_M: DEX combined with mitoTEMPO treatment.

RNA-seq based transcriptome analysis showed that mitoTEMPO treatment led to 1511 genes upregulated and 1653 genes downregulated (Supplemental Figure S3) in DEX rats compared to the vehicle treatment. We analyzed the signal pathways with *p* < 0.05 enriched by KEGG and GO, respectively, and found that some altered signaling pathways in the DEX group were reversed upon mitoTEMPO treatment (Figure 5A). These signaling pathways included blood vessel morphogenesis, angiogenesis, wound healing, cellular response to glucocorticoid stimuli, apoptosis, cellular response to oxidative stress, response to decreased oxygen levels, cellular response to antibiotics, etc. 

GSEA showed that mitoTEMPO treatment significantly affected not only OXPHOS but also glutathione pathways (Figure 5B,C). We then detected the mRNA levels in the glutathione pathway and found that DEX promoted microsomal glutathione S-transferase 2 (Mgst2), glutathione S-transferase theta 1 (Gstt1), and spermine synthase (Sms) mRNA expression, which was reversed by mitoTEMPO treatment, except for Gstt1 (Figure 5D). Given that excess ROS can decrease mtDNA content and subsequently promote mitochondrial dysfunction, we examined the mtDNA content in the placentas. It was found that mtDNA levels were significantly decreased in DEX rats compared to control rats, and this was reversed by mitoTEMPO treatment (Figure 5E). 

The above data indicate that the accumulation of ROS results in a reduced glutathione pathway and the reduction of mtDNA content. Scavenging excess mitochondrial ROS can reverse glutathione pathway and mtDNA content, and it therefore significantly improves the function of the electron transfer chain. 

### 3.7. Impaired Glycolysis and Pentose Phosphate Pathway (PPP) Induced by DEX Might Not Be Associated with Excess ROS

**Figure 6:**
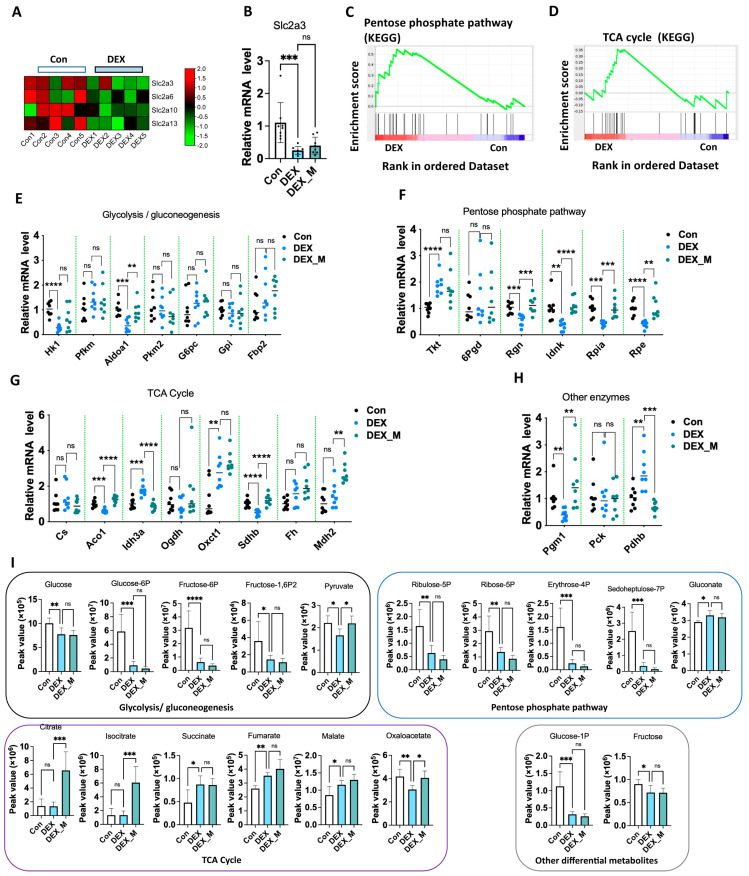
Impaired glycolysis and pentose phosphate pathway (PPP) induced by DEX might not be associated with excess ROS. Pregnant rats were administrated with DEX, DEX combined with mitoTEMPO, or saline from GD7.5 to GD17.5. The rats were sacrificed on GD20.5. (**A**) Heatmap of glucose transporters expression profiles based on the RNA−Seq. (**B**) Analysis of cumulative Q-PCR data of Slc2a3. (**C**,**D**) GSEA analysis showed the signaling related to PPP (**C**) and TCA cycle (**D**). (**E**–**H**) Cumulative data of mRNA levels of gene programs of glycolysis/gluconeogenesis (**E**), PPP (**F**), TCA cycle (**G**), and other enzymes (**H**) by Q−PCR analysis. (**I**) Cumulative data of peak area values of metabolites of central carbon metabolism. * *p*  <  0.05, ** *p*  <  0.01, *** *p*  <  0.001, **** *p*  <  0.0001, ns: no significance. Con: control; DEX_M: DEX combined with mitoTEMPO treatment; Pfkm: phosphofructokinase, muscle; Pkm2: pyruvate kinase M2; G6pc: glucose−6−phosphatase catalytic subunit; GPI: glucose−6−phosphate isomerase; Fbp2: fructose−bisphosphatase 2; 6Pgd: 6−phosphate−glucose dehydrogenase; Cs: citrate synthase; Ogdh: oxoglutarate dehydrogenase; Fh: fumarate hydratase; Pck: phosphoenolpyruvate carboxykinase 1.

Of note, RNA-seq also showed that glucose transporters such as solute carrier family 2 (Slc2a) member 3 (Slc2a3), Slc2a6, Slc2a10, and Slc2a13 were downregulated in DEX rats (Figure 6A). Q-PCR confirmed that Slc2a3 mRNA expression was significantly reduced, and was not reversed by mitoTEMPO treatment (Figure 6B). GSEA analysis showed that the pentose phosphate pathway (PPP) and tricarboxylic acid cycle (TCA cycle) were enriched in the DEX-induced PE model (Figure 6C,D). Given that the PPP pathway is interrelated with glycolytic/gluconeogenesis, and glucocorticoids regulate glycolysis and gluconeogenesis, we then examined the gene program in glycolysis, PPP, TCA cycle, and other enzymes linked to these pathways in the placentas (Figure 6E–H). We conducted a metabolomics analysis targeting central carbon metabolism to elucidate metabolic changes linked to the above pathways (Figure 6I). As shown in Figure 6E–H and Supplemental Figure S4A,B, the mRNA levels of hexokinase 1 (Hk1) and aldolase, fructose-bisphosphate A1 (Aldoa1) in the glycolysis/ gluconeogenesis pathway (Figure 6E) and Slc2a3 mRNA expression (Figure 6B) were significantly decreased in DEX rats compared to control rats. The levels of the metabolites, including glucose, glucose-6P, glucose-1P, fructose, fructose-1,6-P2, and pyruvate were significantly decreased in the DEX group (Figure 6I). MitoTEMPO treatment could reverse reduced Aldoa1 expression, but not the expression of Hk1, the rate-limiting enzyme in glycolysis (Figure 5D). The reduced levels of the above metabolites were not reversed by mitoTEMPO treatment (Supplemental Figure S4). In PPP, regucalcin (Rgn), IDNK gluconokinase (Idnk), ribose 5-phosphate isomerase A (Rpia), and ribulose-5-phosphate-3-epimerase (Rpe) mRNA levels were significantly decreased, whereas transketolase (Tkt) mRNA expression was increased in the DEX group compared to the control group (Figure 6F). The levels of the metabolites, such as erythrose-4P, sedoheptulose-7P, ribose-5P, and ribulose-5P were significantly reduced in the DEX group compared to the control group (Figure 6I). MitoTEMPO treatment reversed the reduction in Rgn, Idnk, Rpia, and Rpe mRNA levels. Still, it did not reverse the increase in Tkt mRNA expression or the reduced levels of the above metabolites (Figure 6F,I. In the TCA cycle and other central carbon metabolism enzymes, aconitase 1 (Aco1), sdhb, and phosphoglucomutase 1 (Pgm1) expression was decreased, while isocitrate dehydrogenase (NAD (+)), 3 catalytic subunit alpha (Idh3a), 3-Oxoacid CoA-transferase 1 (Oxct1), and pyruvate dehydrogenase E1 Subunit beta (Pdhb) mRNA levels were increased in the DEX rats (Figure 6G,H). Succinate, fumarate, and malate were significantly increased, whereas oxaloaletate levels were decreased in the DEX rats compared to control rats (Figure 6I). MitoTEMPO treatment reversed the decreased expression of Aco1, Sdhb, and Pgm1, and increased the expression of Idh3a and Pdhb in the DEX rats. MitoTEMPO treatment could promote malate dehydrogenase 2 (Mdh2) expression in DEX rats (Figure 5G). MitoTEMPO treatment consistently reversed the reduced levels of oxaloaletate, but not those of succinate and fumarate in the DEX group. MitoTEMPO also increased the DEX group’s citrate, isocitrate, and malate levels (Figure 6I). 

The above data suggest that DEX primarily suppresses Hk1 and Slc2a3 expression, and enhances Tkt and Oxct1 expression, while the alternation of other factors in these pathways is attributed to the excess ROS. However, the alternated levels of metabolites in the PPP and the glycolysis pathway were not reversed upon mitoTEMPO treatment, which was due to the reduction of rate-limiting enzyme Hk1 expression, which cannot be reversed by the elimination of excess ROS. 

### 3.8. Excess ROS May Not Contribute to DEX-Induced Alternation of Transcriptional Levels of Placental Cytokines, Prostaglandin Biosynthetic Process and Growth Factors and Increased Circulatory Levels of Proinflammatory Cytokines and PGE2 in DEX-Induced PE Rats

**Figure 7:**
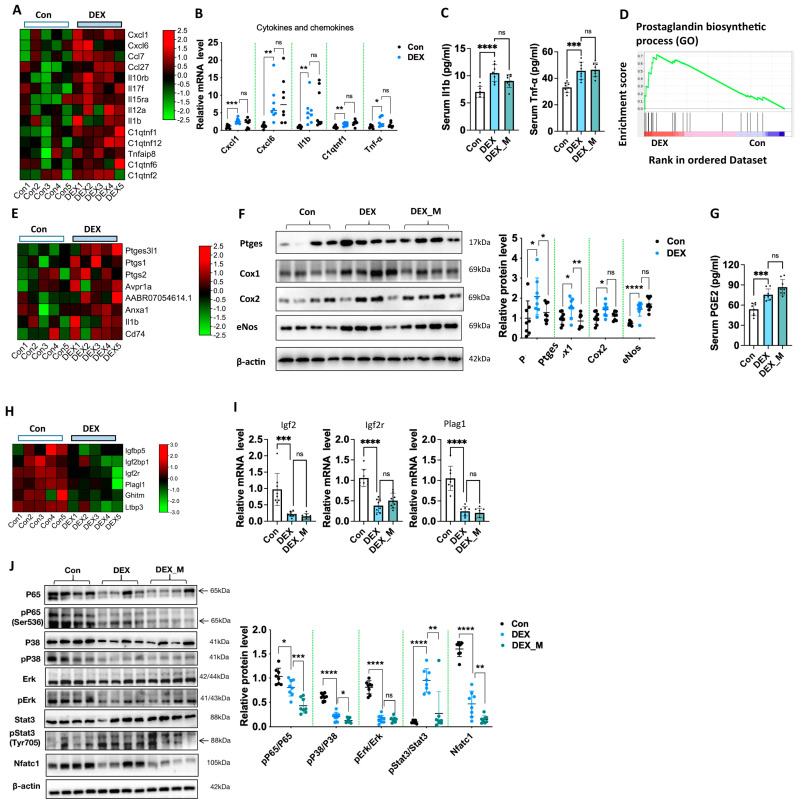
MitoTEMPO treatment could not reverse DEX−induced alternation of transcriptional levels of placental cytokines, prostaglandin biosynthetic process and growth factors, increased circulatory levels of proinflammatory cytokines and PGE2, and changed expression of transcriptional factors in DEX−induced PE model. Pregnant rats were administrated with DEX, DEX combined with mitoTEMPO, or saline from GD7.5 to GD17.5. The rats were sacrificed on GD20.5. (**A**,**B**) Transcriptional levels of cytokines and chemokines. Left panel: Heatmap of cytokines and chemokines expression profiles based on the RNA−Seq (**A**); Right panel: analysis of cumulative Q−PCR data of cytokines and chemokines (**B**). (**C**) Circulatory Il1b and Tnf-α levels. (**D**) GSEA analysis showed the signaling related to the prostaglandin biosynthetic process. (**E**) Heatmap of prostaglandin biosynthetic process−related genes expression profiles based on the RNA−Seq. (**F**) Protein levels of Ptges, Cox1, Cox2, and eNos. Left panel: representative blotting images of Ptges, Cox1, Cox2, and eNos. Right panel: analysis of cumulative data of relative protein level. (**G**) Circulatory PGE2 level. (**H**,**I**) Transcriptional levels of growth factor. Left panel: heatmap of growth factor expression profiles based on the RNA−Seq (**H**); Right panel: analysis of cumulative Q−PCR data of Igf2, Igf2r, and Plag1 (**I**). (**J**) Protein levels of inflammation−related transcriptional factors. Left panel: representative blotting images. Right panel: analysis of cumulative data of relative protein level. * *p*  <  0.05, ** *p*  <  0.01, *** *p*  <  0.001, **** *p*  <  0.0001, ns: no significance. Con: control; DEX combined with mitoTEMPO treatment.

RNA-seq showed that the levels of several chemokines and cytokines were significantly increased (Figure 7A), and GO and KEGG enrichment indicated that cytokines-cytokine receptor interaction occurred in DEX rats (Figure 2A,B). Q-PCR analysis confirmed that Cxcl1, C-X-C motif chemokine ligand 6 (Cxcl6), Il1b, complement C1q tumor necrosis factor-related protein 1 (C1qtnf1), and Tnf-α mRNA levels in placentas were significantly upregulated in DEX rats compared to control rats (Figure 7B). We then determined the levels of proinflammatory cytokines in maternal circulation and found that Il1b and Tnf-α levels were also increased in the DEX group (Figure 7C). MitoTEMPO treatment did not reverse Cxcl1, Cxcl6, Il1b, C1qtnf1 andTnf-α mRNA levels in placentas and Il1b and Tnf-α levels in maternal circulation in DEX rats (Figure 7B,C). These data indicate that the inflammatory state is not associated with mitochondrial oxidative stress in the PE-model caused by maternal DEX exposure. 

GSEA showed that the prostaglandin biosynthetic process was significantly affected in the DEX group (Figure 7D). Transcriptome showed that the mRNA expression of several genes related to prostaglandin synthesis was significantly upregulated (Figure 7E). The protein levels of prostaglandin E synthase (Ptges), cyclooxygenase-1 (Cox1, also known as Ptgs1), and cyclooxygenase-2 (Cox2, also known as Ptgs2) were significantly increased in the DEX group (Figure 7F). PGE2 concentration in maternal circulation was also increased in the DEX group compared with control rats (Figure 7G). Notably, the level of endothelial nitric oxide synthase (eNos), an essential factor in PE development, was upregulated in the DEX group. The protein expression of eNos was significantly increased in DEX rats compared to control rats (Figure 7F). MitoTEMPO treatment did not reverse the increased expression of Cox2 and elevated maternal circulatory levels of PGE2, and increased eNos expression in DEX rats (Figure 7F,G). 

Transcriptome showed that gene programs of regulating fetal growth [30,31,32,33], such as insulin-like growth factor (Igf) associated proteins and their receptors Igf binding protein (Igfbp)5, Igf2 binding protein (Igf2bp)1, Igf2 receptor (Igf2r), pleiomorphic adenoma gene 1 (Plag1) like zinc finger 1 (Plagl1), growth hormone inducible transmembrane protein (Ghitm), and latent transforming growth factor (Ltbp3) were downregulated (Figure 7H). Prior studies have demonstrated that fetal growth can be restricted by a reduced PLAG1-IGF2 pathway [34,35]. Here, we showed that mRNA levels of Plag1, Igf2, and Igf2r were significantly reduced in DEX rats compared to control rats (Figure 7I). MitoTEMPO treatment did not reverse the reduced expression of Plag1, Igf2, or Igf2r (Figure 7I).

Given that JAK/STAT, MAPK, and NF-κB signaling pathways were enriched in the DEX group by GO and KEGG analysis, we determined the levels of transcript factors linked to these pathways. As shown in Figure 7J, the levels of pP65/P65, pErk/Erk, pP38/P38, nuclear factor of activated T Cells 1 (Nfatc1), and pStat3/Stat3 were significantly suppressed in DEX rats compared to control rats. MitoTEMPO treatment could partly reverse pStat3/Stat3 levels but not pP65/P65, pErk/Erk, pP38/P38, or Nfatc1 levels. The above data suggest that DEX primarily inhibits pP65/P65, pErk/Erk, pP38/P38, and Nfatc1 signaling pathways, while DEX upregulates Stat3 signaling via the regulation of ROS production.

### 3.9. DEX Impairment of Human EVTs Function Is Associated with Excess ROS Due to Mitochondrial Dysfunction

**Figure 8:**
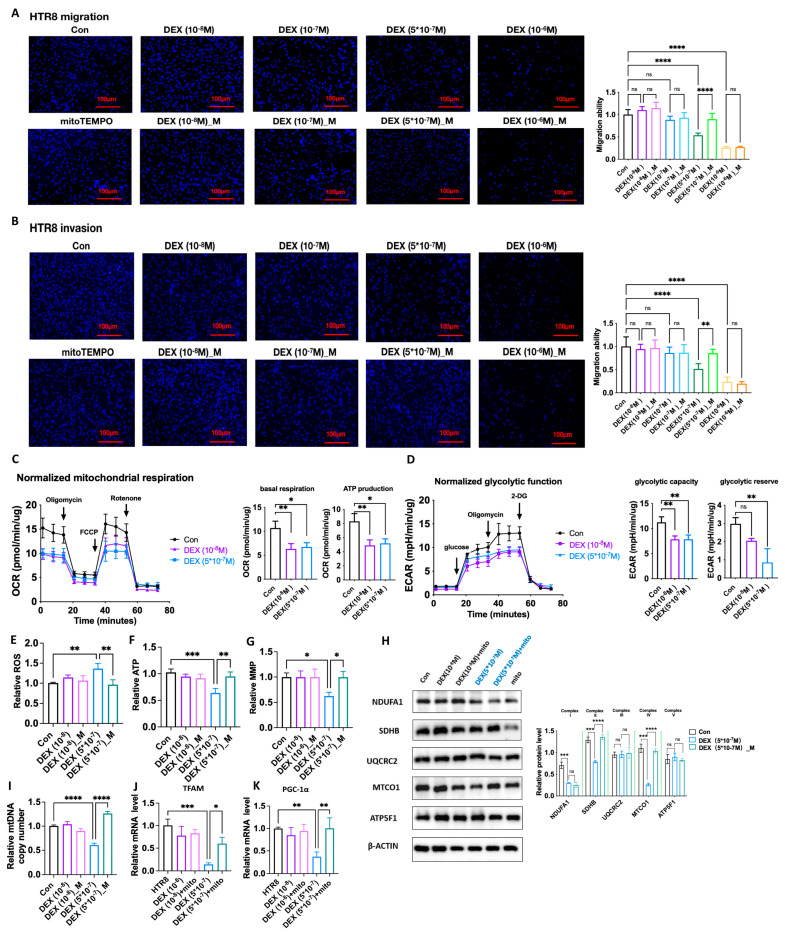
DEX impairment of human EVTs function was associated with regulation of OXPHOS and mitochondrial function. HTR8 cells were treated with DEX (10^−8^ M), DEX (10^−7^ M), DEX (5 × 10^−7^ M), DEX (10^−6^ M), mitoTEMPO (10^−7^ M) or their combination for 24 h. The cells were then used for the migration and invasion analysis as described in Section 2. In some cases, cells were harvested for Q−PCR analysis. (**A**) Analysis of the migration function. Left panel: representative fluorescent images of cells moved to the membrane underside (100×). Right panel: analysis of cumulative migration capacity. (**B**) Analysis of the invasion function. Left panel: representative fluorescent images of cells moved to the membrane underside (100×). Right panel: analysis of cumulative invasion capacity. (**C**) Seahorse mitochondrial respiration assay. Left panel: representative processes of OCR. Right panel: cumulative data of basal respiration and ATP production. (**D**) Seahorse glycolysis stress assay. Left panel: representative processes of ECAR. Right panel: cumulative data of glycolytic capacity and glycolytic reserve. (**E**–**G**) ROS production, ATP and MMP levels. (**H**) Protein levels of NDUFA1, SDHB, UQCRC2, MTCO1, and ATP5F1. (**I**) mtDNA copy number. (**J**,**K**) TFAM and PGC-1α mRNA levels of Q−PCR analysis. n = 3 independent cultures. * *p*  <  0.05, ** *p*  <  0.01, *** *p*  <  0.001, **** *p*  <  0.0001, ns: no significance. Con: control; DEX_M: DEX combined with mitoTEMPO treatment.

Our study showed maternal DEX exposure impaired SA remodeling and trophoblast invasion via the regulation of mitochondrial function in the animal model. We then investigated whether DEX directly impacts trophoblast functions through a modulating mitochondrial function using a cultured human EVTs model. As shown in Figure 8A,B, the DEX treatment dose dependently significantly suppressed the invasion and migration functions in HTR-8/SVneo (HTR8) cells compared with the vehicle treatment. MitoTEMPO (10^−7^ M) treatment could significantly reverse the suppressive effects of DEX (5 × 10^−7^ M) on invasion and migration. 

Oxygen consumption rate (OCR) and extracellular acidification rate (ECAR) analyses showed that basal respiration rate, ATP production and glycolytic capacity were suppressed by DEX treatment (Figure 8C,D). Mitochondrial function analysis showed that ROS production was increased, whilst MMP levels and ATP levels were decreased in the cells with DEX treatment (Figure 8E–G). DEX treatment suppressed NDUFA1 and SDHB, and MTCO1 levels, but not UQCRC2 and ATP5F1 levels (Figure 8H). MtDNA copy number was also reduced in DEX-treated HTR8 cells (Figure 8I). Given that mitochondrial biogenesis is regulated by transcription factor A, mitochondrial (TFAM), and PPAR gamma coactivator 1 alpha (PGC-1α) [36], the mRNA levels of these factors were then examined. As shown in Figure 8J,K, TFAM and PGC-1α mRNA levels were significantly reduced in the cells with DEX treatment compared to the vehicle treatment. 

MitoTEMPO treatment improved mitochondrial functions including ROS production, ATP and MMP levels (Figure 8E–G). Reduced mtDNA copy number and TFAM and PGC-1α mRNA levels caused by DEX could be reversed by mitoTEMPO treatment (Figure 8I–K). Decreased levels of SDHB and MTCO1 caused by DEX could also be reversed by mitoTEMPO treatment, but reduction of NDUFA1 expression induced by DEX was not affected by mitoTEMPO treatment (Figure 8H). These data indicate that DEX primarily inhibits NDUFA1 expression, while reduced SDHB and MTCO1 expression, decreased mtDNA copy number, and decreased TFAM and PGC-1α expression are attributed to ROS accumulation. 

Although placental IL1b and TNF-α mRNA expression was increased in DEX rats, IL1b and TNF-α mRNA expression were suppressed by DEX in HTR8 cells (Supplemental Figure S5). These effects were not affected by mitoTEMPO treatment. 

## 4. Discussion

In the present study, we demonstrated that mitochondrial abnormality was one of the characteristics of the DEX-induced PE model, and excess mtROS production was linked to some PE features, such as maternal hypertension, SA remodeling, and placental blood flow in pregnant rats with DEX exposure. However, it seems that IUGR and elevated sFlt-1 and sEng might not be associated with excess mtROS. 

We found that MitoTEMPO, a mitochondria-targeted ROS scavenger, could alleviate maternal PE-like features, including elevated maternal blood pressure, proteinuria, and kidney morphology. Notably, mitoTEMPO improvement of maternal-fetal perfusion mainly occurred in the maternal side, such as SA and canal blood flow; in contrast, the blood flow of the umbilical artery, the fetal side, was not improved. MitoTEMPO treatment also improved placental hypoxia, which might be attributed to the improvement of placental blood flow. It is known that maternal hypertension is associated with increased resistance to placental blood flow [3,37]. Thus, mitoTEMPO alleviation of maternal hypertension is at least partially associated with improving placental blood flow. In addition, hypertension can cause glomerular injury to a certain extent [38]. Thus, mitoTEMPO improving renal function and morphology might be partly attributed to reduced maternal blood pressure. Nevertheless, the above data suggest that excess mtROS contributes to maternal hypertension and reduced placental blood flow in the DEX-induced PE model. 

It is known that impaired SA remodeling is associated with defects of trophoblast functions [2,3]. Using cultured EVTs, we showed that DEX caused mitochondrial dysfunction and suppressed invasion and migration in EVTs, which was reversed by mitoTEMPO treatment. Taking the data of animal studies and in vitro studies together suggests that DEX suppresses trophoblast functions through detrimental effects on mitochondrial function, and subsequently causes the accumulation of mtROS; and that excess ROS causes impairment of SA remodeling, thereby leading to reduced placenta blood flow and maternal hypertension. 

It is well known that the increased release of angiogenic factors occurs in the placenta with hypoxia, which plays a crucial role in maternal symptoms in PE [39,40]. However, increased levels of anti-angiogenic factors sFlt1 and sEng in maternal blood were not reduced by mitoTEMPO treatment, which may indicate that these factors are not the major players in maternal hypertension in the DEX-induced PE model. In fact, mitoTEMPO treatment only partly improved renal damage in DEX-induced PE rats, and renal injury score in DEX rats with mitoTEMPO treatment was higher than control rats. Some studies have demonstrated that increased sFlt1 causes glomerular injury in pregnant rodent models [41,42]. Thus, suggesting that only partial recovery of renal injury in DEX rats with mitoTEMPO treatment is associated with increased circulatory sFlt1 levels. In addition, we found that vascular branching in the labyrinth zone was less in DEX rats with mitoTEMPO treatment than in controls, although it was improved compared with that in DEX rats. Given that sFlt1 inhibits angiogenesis [43], this indicates that the above phenomena might be also associated with increased circulatory sFlt1 levels. In the present study, however, only a single dosage of mitoTEMPO was used. The effects of higher dosages of mitoTEMPO on the above phenomena require observation in our future studies. 

Many studies have shown that abnormal mitochondrial function can lead to the activation of inflammation [44,45,46], which can also drive mitochondrial dysfunction [47,48]. We found that inflammatory pathways, including the expression of the genes of chemokines and cytokines, as well as prostaglandin synthesis in placentas were increased, and maternal circulatory levels of Il1b, Tnf-α, and PGE2 were elevated in DEX-induced PE model, which could not be reversed by mitoTEMPO treatment. These findings may suggest inflammatory pathways and mitochondrial dysfunction are independent pathways. Since inflammation would be a driving factor of mitochondrial dysfunction, the possibility that mitochondrial dysfunction is caused by inflammation cannot be excluded in the DEX-induced model. 

GCs are known to suppress the expression and activation of transcriptional factors linked to inflammation, such as P65, Erk, and P38 [49,50,51,52]. Consistently, we found that DEX suppressed the transcriptional factors linked to inflammation, such as pP65/P65, pErk/Erk, pP38/P38, and Nfatc1 levels in placentas. Interestingly, proinflammatory cytokines Il1b and Tnf-α mRNA expression in placentas were significantly increased in DEX rats. It is hard to understand such conflicting data between the transcriptional factors and inflammatory cytokines at the current stage. Whether other transcriptional factors are involved in the expression of proinflammatory cytokines and chemokines must be further investigated. However, many studies imply that the conventional anti-inflammatory actions of GCs may not be apparent in the placenta, at least with respect to prostaglandin production [53,54,55,56,57]. Consistent with these studies, the present study showed that Cox1, Cox2, and Ptges expression, and circulatory PGE2 levels were significantly increased in DEX rats. As a proinflammatory mediator, PGE2 can promote the production of proinflammatory cytokines in various tissues [58,59]. Thus, increased circulatory PGE2 levels could increase systemic levels of Il1b and Tnf-α in DEX rats. Although pregnancy is considered a state of low-grade, systemic inflammatory responses [60,61], increased circulatory levels of inflammatory factors including Il1b, Il6, and Tnf-α are associated with adverse pregnancy outcomes, such as PE [62,63,64,65,66]. In addition, inflammatory factors promote the production of antiangiogenic factors sFlt-1 and sEng in placenta [67,68,69]. Some studies have demonstrated that the inhibition of cyclooxygenase and prostaglandin production can relieve placental inflammation, stabilize vascular endothelium, and ultimately alleviate the characterization of PE [70,71]. Taken together, this suggests that increased levels of anti-angiogenic factors in maternal circulation might be associated with a proinflammatory state in the DEX-induced PE model. 

In the present study, we found that DEX exposure could result in IUGR, which is consistent with prior studies [72,73,74]. Many studies have implicated fetal growth restriction caused by DEX exposure as being associated with the detrimental effects of DEX on cell proliferation and differentiation [75,76]. It is known that placental Igfs and Igfbps play important roles in fetal growth [30]. GCs have been shown to suppress the IGF system in various tissues [77,78]. Of note, we found that placental Plag1 and Igf2 expression was reduced in DEX rats, and these effects could not be reversed by mitoTEMPO treatment. As mentioned, fetal growth can be restricted by a reduced PLAG1-Igf2 pathway [34,35]. Thus, it indicates that IUGR caused by DEX exposure is associated with reduced expression of Plag1 and Igf2 in placentas. In addition, it is known that sFlt1 is mainly secreted by syncytiotrophoblasts in human PE placentas [79]. Increased circulatory sFlt-1 is implicated with maternal endothelial dysfunction in PE [80,81]. Kühnel et al. have shown that placental-specific overexpression of sFlt1 results in IUGR in the mouse model [79]. Thus, increased sFlt1 might contribute to IUGR in pregnant rats with DEX exposure. We found that DEX exposure not only caused impaired placental blood flow of the maternal side, but also led to decreased PSV of the umbilical artery. Although mitoTEMPO treatment improved placental blood flow of the maternal side, the placental blood flow of the fetal side could not be improved. Consistently, vasculature branching in the labyrinth zone was reduced upon DEX exposure. Thus, reduced blood flow of the fetal side might also partly contribute to IUGR caused by DEX exposure.

The placenta is highly active in the metabolism to meet the demand of energy and biosynthesis to support the growth of the fetus. Placental metabolic dysfunction is common in PE, and alterations in placental energy metabolism could explicate placental phenotypes of PE, such as decreased placental and fetal growth, redox imbalance, and oxidative stress, etc. [82,83]. The reprogramming of placental metabolism may reflect the dynamic physiological state, enabling it to respond to the stress of PE, while the inability to reprogram placental metabolism may lead to severe PE phenotype. Many studies have shown that glucose uptake and metabolic pathways in the placentas, such as glycolysis, PPP, and TCA cycle have significantly changed in PE. First, the reduction of glucose transport in the placenta is related to PE and fetal growth restriction [84,85,86]. In addition, metabolic reprogramming failure also occurs in PE. Some studies have shown that the activity of glycolytic enzymes is reduced in the placentas of severe PE, leading to impaired glycolytic function, resulting in the reduction of pyruvate and lactate [73,87,88,89]. It has also been shown that the lack or inactivation of 6 phosphate glucose dehydrogenase (6PGD), the key metabolic enzyme of the PPP pathway, is a risk factor for PE [90,91]. It has been shown that IUGR is associated with reduced glucose transporters in placentas [85,86]. Moreover, DEX suppresses the expression of glucose transporters in various tissues [92,93]. The present study found that DEX suppressed the expression of glucose transporter Slc2a3 (glucose transporter 3). Thus, it would lead to a decrease in placental glucose uptake. In addition, we also found that the limiting enzymes of glycolysis and PPP pathways in placentas were inhibited in DEX rats. It suggests that reduced glucose uptake, and inhibited glycolysis and PPP pathways would subsequently lead to the impairment of placental energy metabolism. Of note, the above phenomena were not reversed by mitoTEMPO treatment. As mentioned, placental energy metabolism is critical for fetal growth [84,85]. Collectively, this implies that impaired placental energy metabolism also contributes to IUGR but not to maternal hypertension in the DEX-induced PE model. 

During human pregnancy, the circulatory levels of GCs gradually rise and stay at a stable level (5 × 10^−7^ M) from the midgestational stage to term, then further rise and reach a peak (about 10^−6^ M) at labor [94]. Of note, the dosage of DEX used in the present study was equivalent to about 2–3 × 10^−6^ M in circulation, which is close to GCs levels during the stress state. Thus, our study provides evidence that maternal stress might be a risk factor for PE. Notably, maternal hypertension occurred from GD 9.5 in the rats with DEX exposure. However, nonpregnant rats with DEX treatment at 0.1 mg/kg did not display hypertension or renal injury, which suggests that the organisms are more sensitive to excess GCs during pregnancy than the nonpregnant state because physiological levels of GCs are higher during pregnancy. It is well known that elevated GCs levels can promote blood pressure through GCs’ effects on the cardiovascular system [95]. Some studies have demonstrated that increased GCs levels can promote ROS production in the endothelial cells, which is associated with vasculature constriction [96]. As DEX exposure increased maternal blood pressure as early as GD 9.5, and SA remodeling occurs from GD11.5 to GD13.5 in rats [97], the direct effects of DEX on the maternal cardiovascular system may also contribute to maternal hypertension. It should be pointed out that exogenous GCs (for instance DEX) treatment might affect the level of endogenous GCs in vivo. To better understand the mechanisms underlying the relevant phenomena of pregnant rats with DEX exposure, we must determine the exact levels of corticosteroids and DEX in the circulation and placentas in pregnant rats with DEX treatment. 

GCs can regulate the expression of many genes linked to mitochondria in various tissues [98]. In the present study, we found that DEX treatment primarily inhibited Ndufa1 expression in rat placentas in vivo and cultured human EVTs. It is known that impaired complex I of OXPHOS can lead to mtROS accumulation [99]. MtROS can inhibit PGC-1α expression and subsequently reduce TFAM levels, thereby reducing mtDNA copy [36]. Consistently, we also found that the copy number of mtDNA was reduced in the rat model and cultured EVTs in response to DEX treatment, which could be reversed by mitoTEMPO treatment. In the cultured EVTs, we found that reduced TFAM and PGC-1α expression upon DEX treatment was reversed by mitoTEMPO. These data support that mtROS accumulation plays a crucial role in mitochondrial dysfunction caused by DEX. Interestingly, we found that many molecular pathways caused by DEX treatment were secondarily due to mitochondrial ROS accumulation, as shown in Figure 5 and Figure 6. Thus, the elimination of excess ROS would be a benefit for PE caused by maternal GCs exposure, but it would not improve IUGR. Of note, DEX primarily suppressed Ndufa1 expression in both the animal model and cultured human EVTs. Thus, it would be of interest to investigate the mechanisms underlying DEX regulation of Ndufa1 in trophoblasts. In addition, we found that mitoTEMPO treatment did not reverse the suppressive effects of the high dosage of DEX (10^−6^ M) on the invasion and migration of EVTs, which might be attributed to much more mtROS produced by DEX at 10^−6^ M. Nevertheless, the effects of mitoTEMPO at a higher dosage on the suppressive effects of DEX (10^−6^ M) on migration and invasion should be investigated in the future study. 

There was not consistency in the phenomena of proinflammatory cytokines between the animal model and the human EVT model in response to DEX treatment. For instance, DEX treatment could promote Il1b and Tnf-α mRNA expression in rat placentas in vivo; in contrast, it inhibited IL1b and TNF-α mRNA expression in cultured EVTs. Such discrimination might be attributed to different species used between in vivo and in vitro studies, and might also be associated with the experimental conditions, i.e., in vivo vs. in vitro. However, Il1b and Tnf-α mRNA expression was detected in the labyrinth zone of rat placentas in the DEX model. Thus, the above difference might also be attributed to the different types of trophoblasts examined between in vivo studies and in vitro studies.

We have recently shown that placental 11β-HSD2 dysfunction leads to PE-like features in pregnant rats [13]. It is known that placental 11β-HSD2 dysfunction can cause excess GCs in the placenta. However, the molecular networks are significantly different between the DEX model and the 11β-HSD2 dysfunction model. Compared with the PE model caused by 11β-HSD2 dysfunction [27], more signaling pathways were affected in the DEX-induced PE model, although several common pathways including OXPHOS, thermogenesis, Alzheimer’s disease, and Parkinson’s disease existed in both models (Supplemental Table S3). Several pathways related with inflammation, such as cytokine-cytokine receptor interactions, IL-17 signaling pathways, inflammatory bowel disease (IBD), and positive regulation of JAK-STAT cascade and STAT cascade were significantly enriched in the DEX-induced PE model. Of note, mitoTEMPO treatment can alleviate all the hallmark features of PE, including hypertension, renal damage, IUGR, and increased sFlit-1 and sEng levels in the PE model caused by 11β-HSD2 dysfunction. In contrast, mitoTEMPO treatment partly improved PE features in the DEX model. Such differences in the effects of mitoTEMPO treatment between the two models are attributed to the differences in molecular mechanisms underlying the pathogenesis of the two PE models. PE is a multifactorial disease; a compelling study conducted by Than et al. [100] showed that there are different maternal and placental disease pathways in PE, using an integrated systems biology approach. The maternal disease pathways are reflected in all PE phenotypes, whereas placental disease pathways are superimposed on maternal disease pathways [100,101]. Therefore, it is necessary to take comprehensive consideration to target different molecular groups in each case.

Many studies have demonstrated mitochondrial dysfunction in the placentas of PE patients [102]. Placental mitochondrial dysfunction has also been demonstrated in several animal models of PE, such as the mouse and rat reduced uterine perfusion pressure (RUPP) model [103]. The therapeutic effects of antioxidants targeting mitochondria have also been reported in RUPP models [103,104]. We showed abnormalities in placental mitochondria and the beneficial effects of mitoTEMPO in two PE models, placental 11β-HSD2 dysfunction and maternal DEX exposure. This implies that placental mitochondrial dysfunction is a common feature of PE with various etiologies, and mitochondria-targeted antioxidants have beneficial effects for PE.

The present study has several limitations: (1) Plag1, Igf2, Igfr, and Slc2a3 expression were only determined at the mRNA level; (2) Whether the inflammatory state occurs in placentas upon DEX exposure during pregnancy has not been confirmed; (3) The molecular pathways were only examined in the labyrinth zone of the placentas of DEX rats. The molecular network in the junction zone and decidua requires investigation; (4) Whether increased circulatory sFlt1 and sEng levels are attributed to the increased release of sFlt1 and sEng from placentas requires confirmation; (5) The levels of corticosteroids and DEX in the blood and placentas were not determined in pregnant rats upon DEX treatment.

## 5. Conclusions

In the present study, we demonstrated that maternal GCs exposure leads to PE development, which is associated with large spectrums of molecular pathways affected in the placentas. Maternal GCs exposure impairs mitochondrial function and leads to excess mtROS production in trophoblasts, which leads to impaired SA remodeling, placental ischemia, and subsequently maternal hypertension. However, IUGR caused by DEX might not be associated with SA remodeling. It might be partly due to a reduced IGF system, and impaired energy metabolism in placentas. While increased levels of antiangiogenic factors are not attributed to placental ischemia, they might be related to increased levels of proinflammatory cytokines. Our data gains new insights into the molecular mechanisms underlying PE pathogenesis, in particular, the PE with the etiology of maternal GCs exposure.

## Data Availability

All of the data is contained within the article and the Supplementary Materials.

## Supplementary Materials

The following supporting information can be downloaded at: https://www.mdpi.com/article/10.3390/antiox12050987/s1.
