# Supplementary material for: Mitochondrial ROS Accumulation Contributes to Maternal Hypertension and Impaired Remodeling of Spiral Artery but Not IUGR in a Rat PE Model Caused by Maternal Glucocorticoid Exposure
Source: Antioxidants (Basel). 2023 Apr 24;12(5):987. doi: 10.3390/antiox12050987 (PMC10215507; doi:10.3390/antiox12050987)
Supplement: Supplementary file 1 [file antioxidants-12-00987-s001.zip › antioxidants-2202442-supplementary.pdf]

Online supplement tables and figures

Table S1. The primers for Q-PCR

Primers	Accession Number	Forward Primer (5' -> 3')	Reverse Primer (5' -> 3')
β-actin (Rat)	NM_031144	CGTAAAGACCTCTATGCCAACA	TAGGAGCCAGGGCAGTAATC
Mgst2 (Rat)	XM_039102007	TTCAATCAAGTTTTTGCAACC	TCTTGGCAACATGAAAGTCC
Gstt1 (Rat)	XM_039098444	CCAGTCTTTGAAGGGCGTCC	GGGCGCACAGTCGTGTAATG
Sms (Rat)	XM_032889689	GGATTGGTATTGCTGGACCT	CCAAATTAACATCCCCGCTG
Rps18 (Rat)	XM_032888536	TCTTCCACAGGAGGCCTACA	ACAGCAAAGGCCCAAAGACT
16SrRNA (Rat mtDNA specific)	KP244683	AATTGCCATGGCCTTCCTCA	TATGGCTATTGGTCAGGCGG
Hk1 (Rat)	XM_032888314	CGGAATGGGAGCCTTTGG	GCCTTCCTTATCCGTTTCAATGG
Pfkm (Rat)	XM_032885836	GAGCGAGAAGGACGACTCC	GCCTCCGATGACACACAGA
Aldoa1 (Rat)	XM_032892639	CACTACTCAAGGGCTGGACG	CCCACCCCAAGCCTTTAGAG
Pkm2 (Rat)	XM_032909251	CGCCTGGACATTGACTCTTG	GAAATTCAGCCGAGCCACATT
G6pc (Rat)	XM_032912859	TGCTCCACGACTTTGGGATC	ATGCCTGACAAGACTCCAGC
Gpi (Rat)	XM_032893879	TCAAGCTGCGCGAACTTTTTG	GGTTCTTGGAGTAGTCCACCAG
Fbp2 (Rat)	XM_032885034	ACCCTGACCCGTTACGTTATG	ACATTCACGCTCCCCGAAATC
Tkt (Rat)	XM_032918543	CTGGAAGACCTGGCCATGTT	TTCACAGCTTGCACAATGGC
6Pgd (Rat)	XM_032894485	ATGGCCCAAGCTGACATTG	GCACAGACCACAAATCCATGAT
Rgn (Rat)	XM_032889872	TCAGTGGCACTTCGACAGTC	ATCCTTCCCTCCAAAGCAGC
Idnk (Rat)	XM_032885000	CCCATCAGCTCCCGAAAACT	AGTACACCGCAGCTGTCTTC
Rpia (Rat)	XM_032906334	CTTTGAGTCTCCAGCCCAGG	TACAAACCCGACTGCTCCAC
Rpe (Rat)	XM_032901291	GAAGCGGCTCAGAAACGTTC	CATGCTACCCCACCAAACCT
Cs (Rat)	XM_039078325	GGACAATTTTCCAACCAATCTGC	TCGGTTCATTCCCTCTGCATA
Aco1 (Rat)	NM_017321 XM_346735	TTGACGAACAGAGGCCTGAC	GCACCTACTGGGCCATCTTT
Idh3a (Rat)	XM_032911558	TGGGTGTCCAAGGTCTCTC	CTCCCACTGAATAGGTGCTTTG
Ogdh (Rat)	XM_032916969	GGTCCACCAGGTTTGAGGAG	GTTGACAACCACGTGAACGG
Oxct1 (Rat)	XM_032898826	TGCCAATGCAGCAGATCTCA	TGGTAGAGCCTATGCCCTGT
Sdhb (Rat)	XM_032895252	GGAGGGCAAGCAACAGTATC	TTGTCTCCGTTCCACCAGTAA
Fh (Rat)	XM_032915007	AGAATCTACGAGCTCGCTGC	GTGTTGGCCTGGATCCCTAC
Mdh2 (Rat)	XM_032886508	AGGAAGCCAGGAATGACACG	TTTTCCCCAACAGCAAGGGT
Slc2a3 (Rat)	NM_001412552	CACTGTGCTGGAGCTCTTCA	CAGCCGAGGGGAAGAACATT
Pgm1 (Rat)	XM_032901777	AGCGAGTGAAGGTGTTCCAG	GCATAGGCCTCGACTGAGTC
Pck (Rat)	XM_032904709	GTAGGAGCCGCCATGAGATC	TGCTGGTTTGCTCCTAGCTC
Pdhb (Rat)	XM_032918316	AGGAGGGAATTGAATGTGAGGT	ACTGGCTTCTATGGCTTCGAT
Cxcl1 (Rat)	NM_030845	GCAGACAGTGGCAGGGATTC	CGACCATTCTTGAGTGTGGCTAT
Cxcl6 (Rat)	XM_032916829	TGATCCCTGCAGGTCCACA	GGCTGATCTGACCAGTGCAA
Il1b (Rat)	XM_032902343	TGCAGGCTTCGAGATGAA	CCGACCATTGCTGTTTCC
C1qtnf1 (Rat)	XM_006247801	GTGGTCTTCGACACGGAGTT	TGATCTCATCCATCGGCAGC
Tnf-α (Rat)	NM_012675	TGCCTCAGCCTCTTCTCATT	TGGTATGAAGTGGCAAATCG
lgf2 (Rat)	XM_032891370	AGGGGAGCTTGTTGACACG	GGGTATCTGGGGAAGTCGTC
lgf2r (Rat)	XM_032894713	ATAAACCAAGAAATTCTGCGGG	GGGTGACTTACAGACACCGTGA
Plag1 (Rat)	XM_032907808	ATGGCCACTGTCATTCCTGG	TGCGTGAGATTTCAGGTGCT
β-actin (Human)	NM_001101	GGCACCCAGCACAATGAAG	CCGATCCACACGGAGTACTTG
B2M (Human)	XM_054378457	TGTTCCTGCTGGGTAGCTCT	CCTCCATGATGCTGCTTACA
16SrRNA (Human mtDNA specific)	OQ707211	GGTGCAGCCGCTATTAAAGG	ATCATTTACGGGGGAAGGCG
TFAM (Human)	NM_003201	GCGTTTCTCCGAAGCATGTG	TCTTCAGCTTTTCCTGCGGT
PGC-1α (Human)	XM_054348813	AAGGATGCGCTCTCGTTCAA	TTCGTTTGACCTGCGCAAAG
Tnf-α (Human)	NM_000594	CCTCTCTCTAATCAGCCCTCTG	GAGGACCTGGGAGTAGATGAG
Il1b (Human)	NM_000576	ATGATGGCTTATTACAGTGGCAA	GTCGGAGATTCGTAGCTGGA

Table S2. Antibodies for western blotting

Antibody	Manufactory	Catalog Number	Host / Isotype
Ndufa1	Boster	BA3676	Rabbit / IgG
Sdhb	Proteintech	10620-1-AP	Rabbit / IgG
Uqcrc2	Proteintech	14742-1-AP	Rabbit / IgG
Mtco1	Immunoway	YN0177	Rabbit / IgG
Atp5f1	Proteintech	15999-1-AP	Rabbit / IgG
Hif1a	Abcam	ab210073	Rabbit / IgG
Ptges	Abcam	ab180589	Rabbit / IgG
Cox1	CST	9896S	Rabbit / IgG
Cox2	Proteintech	12375-1-AP	Rabbit / IgG
eNos	Proteintech	27120-1-AP	Rabbit / IgG
P65	Immunoway	YM3111	Mouse / IgG
pP65 (Ser536)	Immunoway	YP0191	Rabbit / IgG
P38	Boster	BM4439	Rabbit / IgG
pP38 (Thr180+Tyr182)	Bioss	bs-2210R	Rabbit / IgG
Erk1/2	Immunoway	YT1625	Rabbit / IgG
pErk1/2	HUABIO	ET-1610-13	Rabbit / IgG
Stat3	Proteintech	10253-2-AP	Rabbit / IgG
pStat3 (Tyr705)	Immunoway	YP0251	Rabbit / IgG
β-actin	Proteintech	20536-1-AP	Mouse / IgG

Table S3. Comparison of DEX-induced and 11β-HSD2 dysfunction-induced PE models

Molecular Pathways and Phenotypes		PE model	
**Phenotypes response to mitoTEMPO**	**Enrichment pathways**	**DEX**	**11β-HSD2 dysfunction**
	Oxidative phosphorylation	+	+
	Thermogenesis	+	+
	Alzheimer disease	+	+
	Parkinson disease	+	+
	cytokine-cytokine receptor interactions	+	−
	IL-17 signaling pathways	+	−
	inflammatory bowel disease (IBD)	+	−
Hypertension		+	+
Renal Injury		+	+
sFlt1, sEng...		−	+
IUGR		−	+

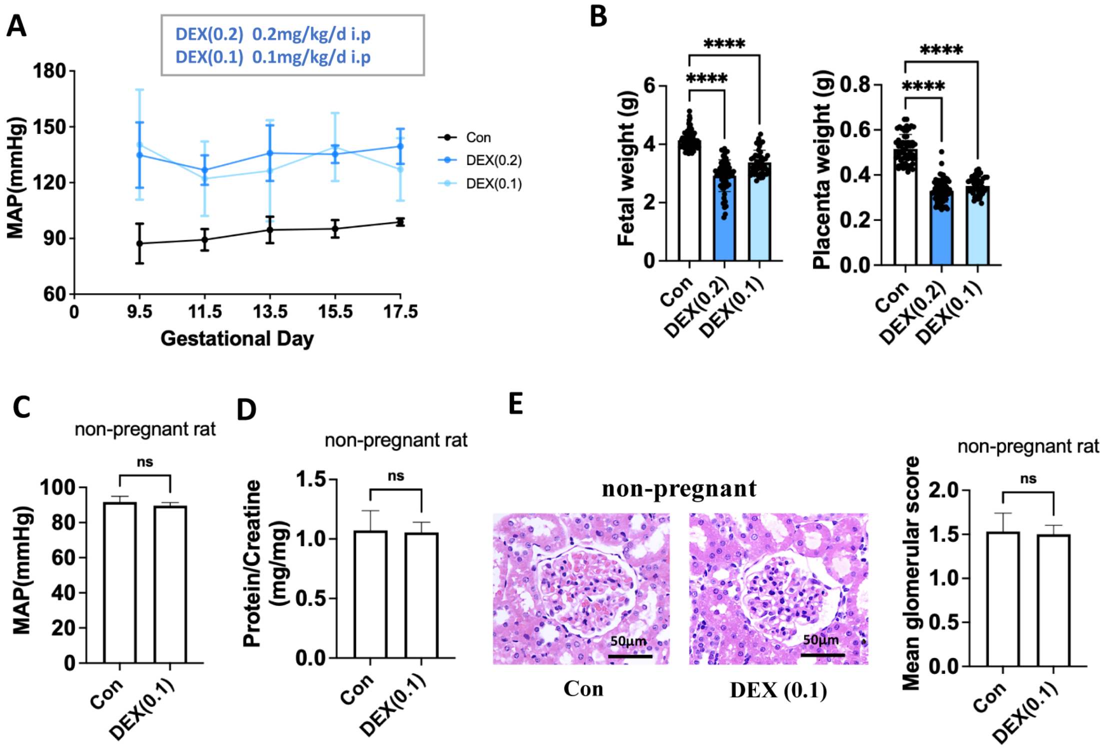

Figure S1. The effects of DEX on blood pressure in pregnant rats and nonpregnant rats and placental and fetal growth in pregnant rats. Pregnant rats were administrated with DEX at 0.1mg/kg or 0.2mg/kg or saline once a day from GD7.5 to GD17.5 and sacrificed on GD20.5. Rats of Con group received same volume of saline. Nonpregnant female rats were randomly divided into two groups Con or DEX (n=5 in each group). The rats were injected saline or DEX at 0.1mg/kg once a day for 10 days. Rats of Con group received same volume of saline. The urine was collected on the last day of injection. A, MAP measured from GD 9.5 until GD 17.5. B, fetal and placental weight from 8 dams (each group) measured on GD 20.5. It represented as individual fetal or placental weight. C, MAP measured on second day of the last injection. D, protein/creatinine (mg/mg) in urine. E, morphology of glomeruli stained by H&E and PAS. Left panel: the representative images (400×). Right panel: histopathological score of glomeruli. ****$p < 0.0001$. Con: control; MAP: mean arterial pressure.

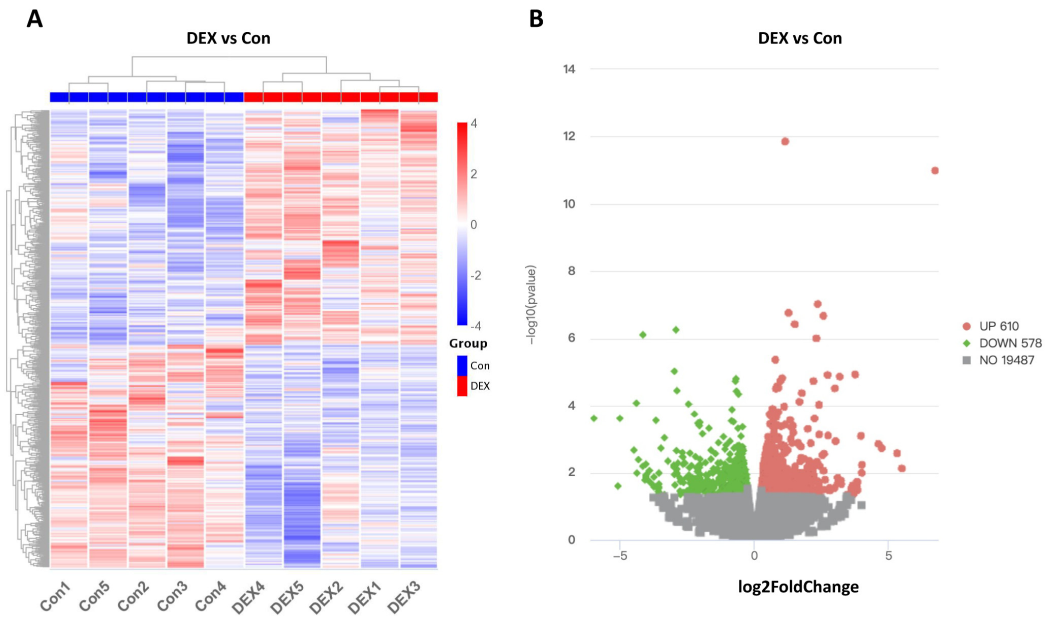

Figure S2. The heatmap and volcano plot of RNA sequencing-based transcriptome analysis of the placentas in DEX-induced PE model. Pregnant rats were administrated with DEX or saline from GD7.5 to GD17.5. The placentas were collected on GD20.5 and placental tissues were collected for RNA-seq. A, cluster heat map of the differential genes. B, volcano plot of differential genes in RNA-Seq between DEX and controls. Con: control.

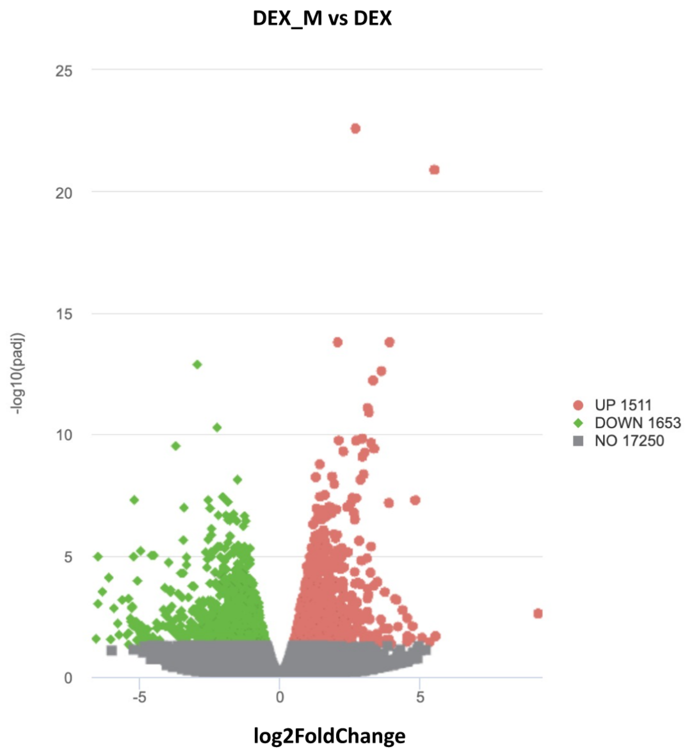

Figure S3. Volcano plot of differential expressing genes in RNA-Seq among DEX rats and DEX rats with mitoTEMPO treatment. Pregnant rats were administrated with DEX, DEX combined with

mitoTEMPO from GD7.5 to GD17.5. The rats were sacrificed on GD20.5 and the placental tissues were collected for RNA-seq. DEX_M: DEX combined with mitoTEMPO treatment.

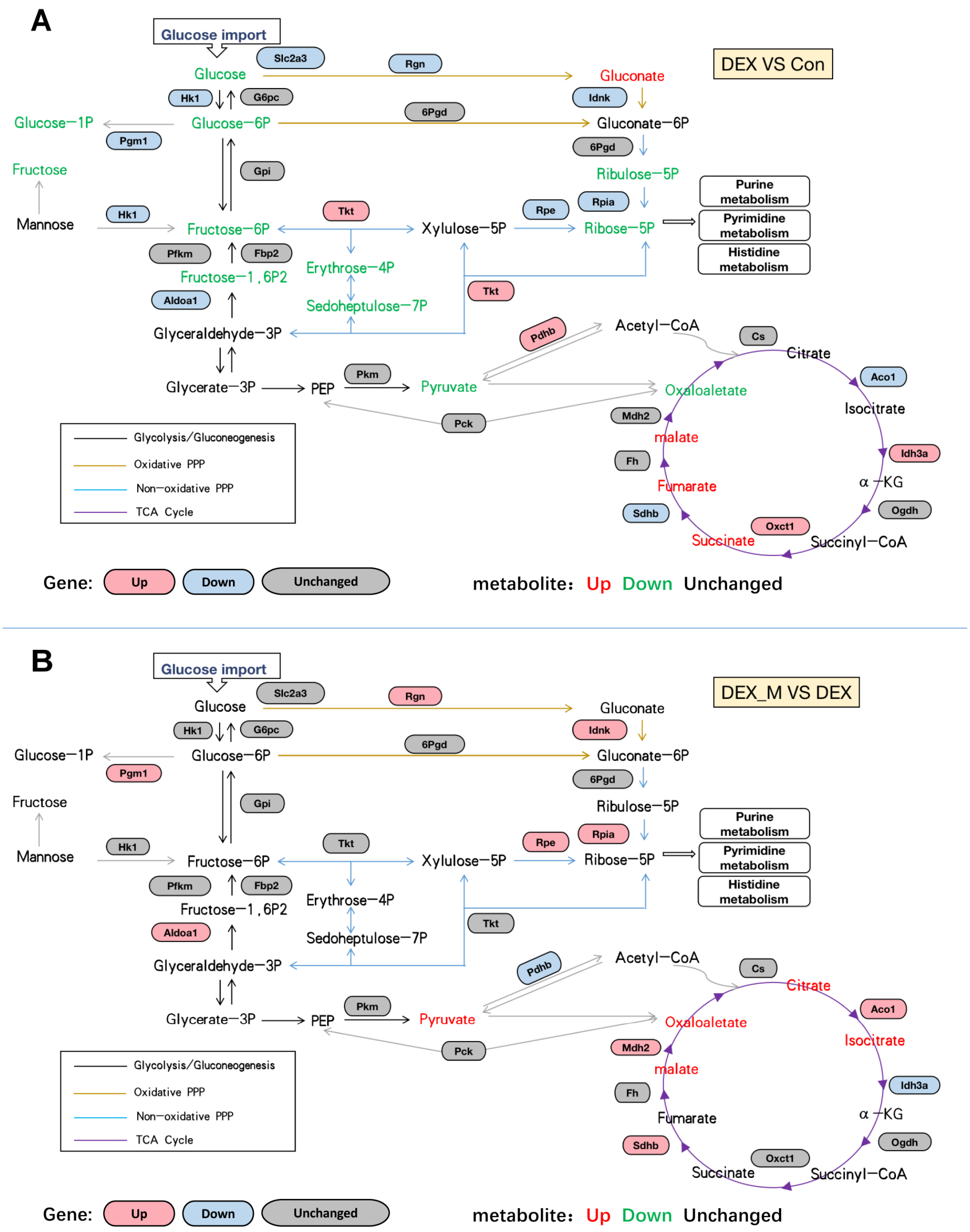

Figure S4. The map of central carbon metabolism pathways in placentas of DEX versus Con (DEX vs Con) and DEX_M versus DEX (DEX_M vs DEX). Pregnant rats were administrated with DEX (A), DEX combined with mitoTEMPO (B) from GD7.5 to GD17.5. The rats were sacrificed on GD20.5 and the placental tissues were collected. Blue and green colors indicate reduced genes and metabolites, red indicates increased genes and metabolites. DEX_M: DEX combined with

mitoTEMPO treatment.

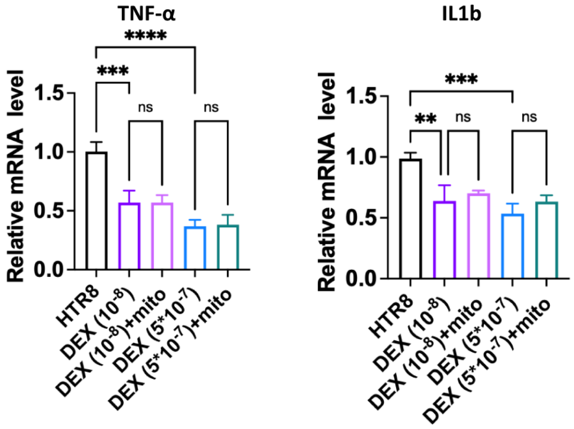

Figure S5. The effects of DEX and DEX combined with mitoTEMPO on TNF-α and IL1b mRNA level in cultured HTR8 cells. HTR8 cells were treated with DEX (10^{-8}M), DEX ($5*10^{-7}$M), DEX (10^{-8}) with mitoTEMPO (10^{-7}M) or DEX ($5*10^{-7}$) with mitoTEMPO (10^{-7}M) for 24h. The cells were harvested for determination of TNF-α and IL1bmRNA expression. n=3 independent cultures. **$p < 0.01$, ***$p < 0.001$, ****$p < 0.0001$. Con: control; DEX_M: DEX combined with mitoTEMPO treatment
